# Reconstruction of cobalt magnesium aluminum hydrotalcite loaded ruthenium using the memory effect for selective oxidation of 5-hydroxymethylfurfural under alkali-free conditions†

**DOI:** 10.1039/d5ra02352a

**Published:** 2025-07-18

**Authors:** Shuang Zhang, Sai Wang, Ji Ma, Suzhen Cao

**Affiliations:** a Institute of Petrochemical Technology, Jilin Institute of Chemical Technology 45 Chengde Street Jilin 132022 PR China zs3062332@126.com

## Abstract

The hydrotalcite CoMgAl-LDH was prepared using a hydrothermal method. The calcined hydrotalcite was rehydrated utilizing its “memory effect”, and the catalyst Ru/CoMgAl-RLDH was prepared by loading ruthenium nanoparticles through the impregnation reduction method. The pristine hydrotalcite Co_1_Mg_2_Al_1_-LDH exhibited a specific surface area of 9.2 m^2^ g^−1^, while the Ru-loaded reconstructed hydrotalcite Ru_4_/Co_1_Mg_2_Al_1_-RLDH demonstrated a significant increase in specific surface area to 130.0 m^2^ g^−1^. The total basicity of the original hydrotalcite Co_1_Mg_2_Al_1_-LDH catalyst surface was 0.25 mmol g^−1^, and all these basic sites were weak basic sites. By contrast, the total basicity of the reconstructed hydrotalcite Ru_4_/Co_1_Mg_2_Al_1_-RLDH catalyst surface was 0.37 mmol g^−1^, consisting of weak and moderate basic sites. The reconstructed hydrotalcite not only showed higher thermal stability than the original one but also enabled the systematic adjustment of its basic sites by changing the molar ratio of elements in the hydrotalcite and the calcination temperature. The Ru_4_/Co_1_Mg_2_Al_1_-RLDH catalyst prepared with the Co : Mg : Al molar ratio of 1 : 2 : 1, calcination temperature of 400 °C, and Ru loading of 4 wt% demonstrated optimal catalytic activity, achieving complete 5-hydroxymethylfurfural (HMF) conversion and 87.6% 2,5-furandicarboxylic acid (FDCA) yield under alkali-free conditions.

## Abstract 2

The hydrotalcite CoMgAl-LDH was prepared using a hydrothermal method.

## Graphical abstract

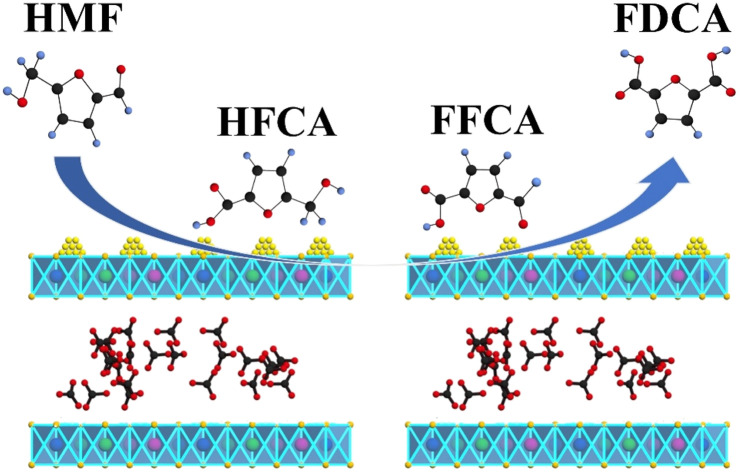

## 1. Introduction

In recent years, the conversion of renewable platform chemicals into valuable commercial chemicals through selective oxidation has garnered considerable attention within the catalysis community. 5-Hydroxymethylfurfural (HMF) can be selectively oxidized to generate a variety of useful downstream chemicals, such as 2,5-difuroic acid (DFF), 5-hydroxymethyl-2-furancarboxylic acid (HFCA), 5-formyl-2-furancarboxylic acid (FFCA), and 2,5-furandicarboxylic acid (FDCA). These products are widely utilized in the manufacture of novel polymers, pharmaceuticals, and other high-value fine chemicals.^1,2^ HMF can be considered a promising biomass platform compound. As one of the downstream products of HMF oxidation, FDCA is a key monomer for the production of polyethylene furanoate (PEF), which is as an emerging ideal alternative to traditional petroleum-based polyethylene terephthalate (PET). PEF finds applications in the production of packaging materials, soft drink bottles, films, and fibers.^3^ Therefore, FDCA is a versatile building-block chemical.

It is well known that the selective oxidation of HMF to FDCA involves multiple sequential steps, including the oxidation of both the hydroxymethyl and aldehyde functional groups, which imposes stringent requirements on the structural design and performance of catalysts. In the reaction of HMF oxidation catalyzed by alkali to synthesize FDCA, the addition of homogeneous alkali can enhance the yield of FDCA. However, from an industrial perspective, excessive homogeneous alkali may induce undesirable HMF degradation through base-catalyzed side reactions, increasing the cost of separation steps.^4^ Therefore, designing solid base catalysts that can replace homogeneous alkali is of great significance for the efficient and selective oxidation of HMF to FDCA. Hydrotalcite is a common solid base catalyst that exhibits high activity and selectivity in alkali-driven reactions as a substitute for homogeneous alkali.^5^ Hydrotalcite is a type of anionic clay mineral with layered double hydroxides (LDHs), it consists of positively charged main layers with metal hydroxides and exchangeable interlayer regions with compensating anions and solvated molecules. The general formula of layered double hydroxides can be written as [M_1−*x*_^2+^M_*x*_^3+^(OH)_2_][A^*n*−^]_*x*/*n*_·*m*H_2_O, where M^2+^ and M^3+^ correspond to divalent and trivalent metallic cations respectively, A^*n*−^ represents the intercalated anionic species, the parameter *x* denotes the molar fraction of trivalent cations (usually 0.2 < *x* < 0.33), and *m* is defined as the number of solvated molecules (usually water).^6^ The alkali strength of hydrotalcite can be controlled by adjusting the properties of metal ions and preparation methods. The metal elements in its layered plates are adjustable, and the interlayer anions are exchangeable. This structural tunability endows hydrotalcite with flexibility in various catalytic reactions, allowing optimization according to different reaction requirements.^7^

Hydrotalcite, with its diverse structure, has been widely used as a catalyst support. It was reported that Li *et al.* synthesized the catalyst, AuPd/LaCaMgAl-LDH, by loading Au and Pd onto La-doped CaMgAl layered double hydroxide (LDH) support. Under the reaction conditions of 120 °C, 6 h, and 0.5 MPa O_2_, complete HMF conversion was achieved with 99% selectivity toward FDCA. The results indicated that the synergistic effect of bimetallic Au and Pd nanoparticles, along with the basicity of the LaCaMgAl-LDH support surface, played a significant role in enhancing catalytic performance.^8^ Ebitani *et al.* prepared PdPt-PVP loaded onto magnesium–aluminum hydrotalcite (HT) using polyvinyl pyrrolidone (PVP). Under the reaction conditions of 95 °C, 11.5 h, and an O_2_ flow rate of 40 mL min^−1^, the conversion rate of HMF reached 100%, with selectivity to FDCA of 99%.^9^ Additionally, some non-noble metals, like the transition metal cobalt, also display high activity during the selective catalysis of HMF.^10^ Gao *et al.* prepared CoFeS and CoFeP-400 catalysts through hydrothermal sulfidation and phosphation of CoFe-LDH. Under the reaction conditions (acetonitrile as solvent, reaction temperature of 120 °C, t-BuOOH as oxidant, and reaction time of 12 h), the conversion rate of HMF reached 100% for both catalysts, while the yields of FDCA were 87.1% and 89.5% respectively.^11^

Another notable characteristic of hydrotalcite is its ability to be transformed into mixed metal oxides (LDO) through calcination. These mixed metal oxides, utilizing their “memory effect,” can revert to the ordered layered hydrotalcite structure through rehydration.^12^ The physicochemical properties of the reconstructed hydrotalcite vary depending on its structure. The specific surface area of the reconstructed hydrotalcite exceeds that of the original hydrotalcite, offering more active sites and enhancing catalytic activity.^13^ The rehydrated hydrotalcite exhibits higher thermal stability than the original one, which is crucial for reactions requiring operation at elevated temperatures.^14^ Through thermal treatment and rehydration, the basic sites of hydrotalcite can be systematically adjusted.^15^ Xia *et al.* loaded a series of bimetallic Pd–Au nanoparticles with varying proportions onto the reconstructed hydrotalcite, preparing the catalyst Pd–Au/HT. Under the reaction conditions (Au/Pd ratio of 4, O_2_ flow rate at 60 mL min^−1^, addition of NaOH as base, and reaction temperature of 60 °C for 6 h), complete conversion of HMF was achieved, with yield of FDCA exceeding 90%. The synergistic and electronic effects between Au and Pd facilitate the oxidation reaction.^16^ However, in its reaction conditions, both homogeneous alkali and solid alkali were added simultaneously, which increased the cost, and the homogeneous alkali affected the subsequent separation of FDCA. The calcination temperature of this hydrotalcite was 550 °C. During the reconstruction process, the excessively high calcination temperature had an impact on the restoration of the hydrotalcite to its layered structure.^17^

In the reported literature on hydrotalcite as support, most studies utilize expensive precious metals such as Pd,^18^ Au,^19^ and Pt.^20^ Ru,^21^ being more cost-effective compared to other precious metals and possessing the more flexible valence state (−2 to +8), this study for the first time selected it as the noble metal active component supported on Co-doped magnesium–aluminum hydrotalcite. This approach facilitated the selective oxidation of HMF to FDCA in an alkali-free aqueous solution. First, the reconstruction of hydrotalcite using the memory effect as a support for ruthenium loading enriched the basic sites of the catalyst and exhibited higher thermal stability. Second, the doping of cobalt improved the activity of the catalyst. The Ru_4_/Co_1_Mg_2_Al_1_-RLDH catalyst, prepared with the Co : Mg : Al molar ratio of 1 : 2 : 1, the calcination temperature of 400 °C, and the Ru loading of 4 wt%, demonstrated the highest activity. HMF was completely converted, with the yield of FDCA reaching 87.6% under alkali-free conditions. Through characterization analysis of the catalyst, the reaction mechanism was explored and compared with those of other noble metal-supported hydrotalcite catalysts and homogeneous alkali catalytic systems. This work provides insights for the rational design of reconstructed hydrotalcite for selective oxidation reactions in alkali-free system.

## 2. Experimental

### 2.1. Preparation of the catalyst

A total of 12 mmol of Mg(NO_3_)_2_·6H_2_O, Co(NO_3_)_2_·6H_2_O, and Al(NO_3_)_3_·9H_2_O were dissolved in 30 mL of pure water according to different molar ratios (0.5 : 2.5 : 1, 1 : 2:1, 1.5 : 1.5 : 1, 2 : 1 : 1, 2.5 : 0.5 : 1) to obtain a mixed salt solution. Simultaneously, a solution was prepared by dissolving 24 mmol of urea in 30 mL of pure water. Under stirring conditions, the urea solution was slowly dropped into the mixed salt solution. The reaction vessel containing the mixture was hydrothermally treated at 120 °C for 24 h. Once the reaction kettle had cooled naturally to the ambient temperature, the mixture within was carefully subjected to centrifugation, followed by filtration. Subsequently, the filter residue underwent repeated washings with deionized water to comprehensively eliminate any residual impurities. Finally, the resultant precipitate was meticulously transferred to an oven set at the consistent temperature of 80 °C and left for the duration of 12 h, facilitating the thorough evaporation of all moisture. The obtained material was labeled as CoMgAl-LDH. The CoMgAl-LDH was placed into a muffle furnace and calcined at 400 °C, resulting in the formation of CoMgAl-LDO. Additionally, the CoMgAl-LDH was separately subjected to high-temperature calcination at 300 °C and 500 °C, which were denoted as CoMgAl-LDO (300) and CoMgAl-LDO (500), respectively.

Weighed a certain amount of RuCl_3_·3H_2_O (with Ru accounting for 1–5 wt% of the support mass fraction) and dissolved it in 12.5 mL of pure water. Added 0.5 g of support to the solution, and under stirring conditions, immersed and dispersed it for 12 h while rehydrating. Weighed NaBH_4_ according to the molar ratio of BH_4_^−^ in NaBH_4_ to Ru^3+^ in RuCl_3_·3H_2_O, which was 20 : 1, and dissolved it in a 0.5 wt% NaOH aqueous solution to achieve a NaBH_4_ concentration of 1.5 mol L^−1^. Then, added it dropwise to the dispersion of ruthenium trichloride and support, stirred, and reduced it for 12 h. Filtered and washed the mixed solution until it was neutral, and dried it under vacuum for 12 h to obtain catalysts loaded with different amounts of Ru, denoted as Ru_*z*_/CoMgAl-RLDH (*z* = 1,2,3,4,5).

### 2.2. Catalytic reaction equipment and product detection

A certain amount of catalyst, deionized water (5 mL), and HMF (0.2 mmol) were added to the high-pressure reactor. After the reaction vessel was installed, introduced oxygen (0–1 MPa) into the vessel, set the magnetic stirring speed to 500 rpm, heat up to a certain temperature and maintain it for the certain period of time. After the reaction was complete, stop heating and allow the temperature to drop to room temperature. Then, exhaust the gas and filter out the catalyst. The diluted reaction solution was analyzed using an Agilent 1260 Infinity II high-performance liquid chromatograph to calculate the conversion rate of HMF and the yield of FDCA. Liquid phase testing conditions: UV detector. Poroshell 120 (3.0 mm × 150 mm, 2.7 μm) C18 chromatographic column was selected, 0.1 wt% formic acid aqueous solution: acetonitrile = 95 : 5 is used as the mobile phase, the mobile phase ratio starts to change at 0.5 min and reaches 0.1 wt% formic acid aqueous solution: acetonitrile = 60 : 40 at 4.5 min, the column temperature is set at 30 °C, the injection volume is set at 5 μL, the peak position and peak area are recorded, and the external standard method is used for quantitative analysis of the product.

## 3. Results and discussion

### 3.1. Structure of catalysts

**Fig. 1:**
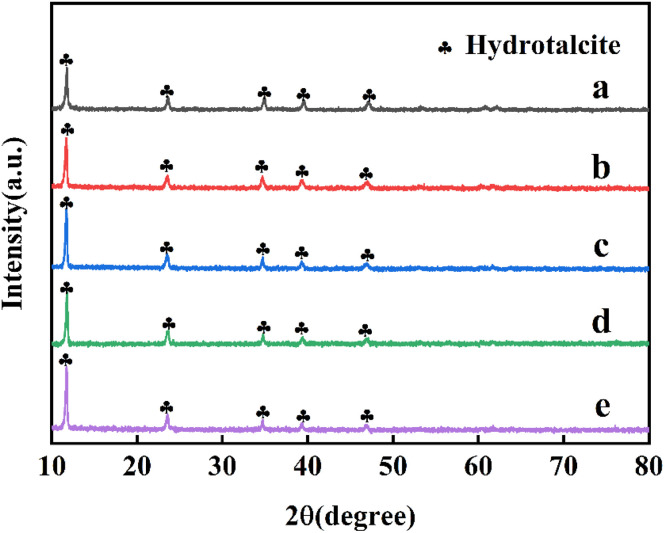
XRD patterns of (a) Co_0.5_Mg_2.5_Al_1_-LDH, (b) Co_1_Mg_2_Al_1_-LDH, (c) Co_1.5_Mg_1.5_Al_1_-LDH, (d) Co_2_Mg_1_Al_1_-LDH, (e) Co_2.5_Mg_0.5_Al_1_-LDH.

**Fig. 2:**
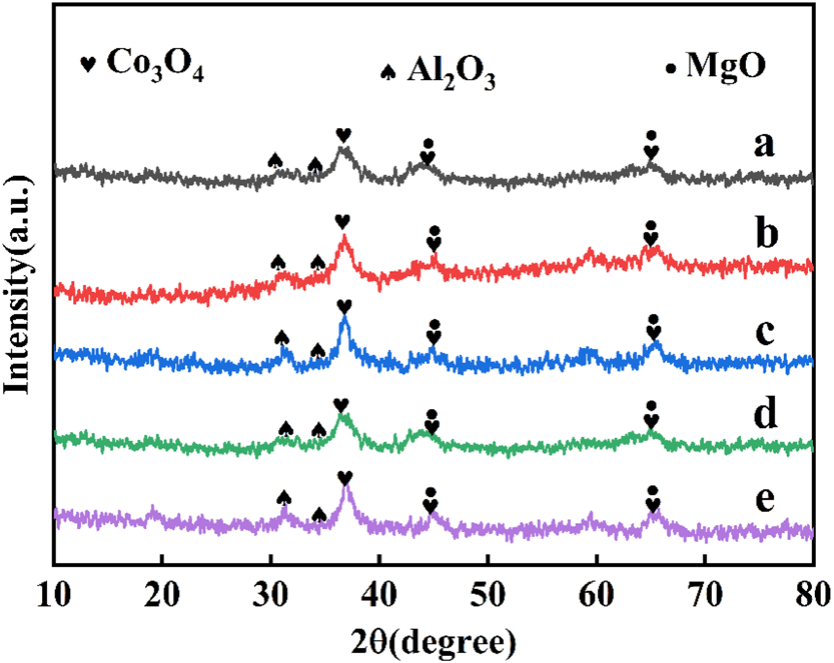
XRD patterns of (a) Co_0.5_Mg_2.5_Al_1_-LDO, (b) Co_1_Mg_2_Al_1_-LDO, (c) Co_1.5_Mg_1.5_Al_1_-LDO, (d) Co_2_Mg_1_Al_1_-LDO, (e) Co_2.5_Mg_0.5_Al_1_-LDO.

**Fig. 3:**
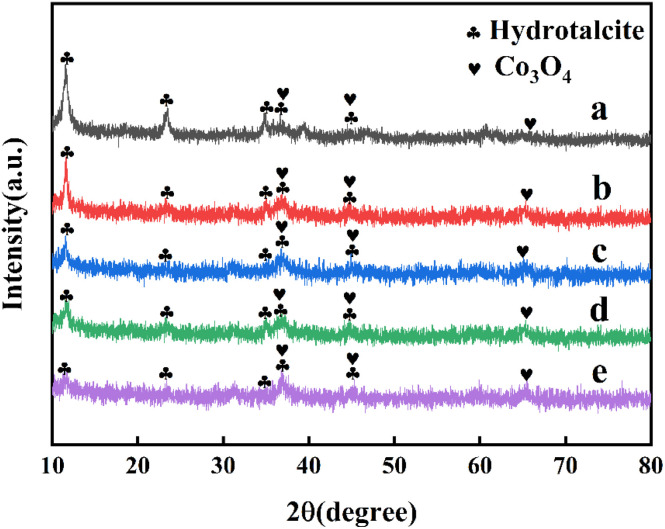
XRD patterns of (a) Co_0.5_Mg_2.5_Al_1_-RLDH, (b) Co_1_Mg_2_Al_1_-RLDH, (c) Co_1.5_Mg_1.5_Al_1_-RLDH, (d) Co_2_Mg_1_Al_1_-RLDH, (e) Co_2.5_Mg_0.5_Al_1_-RLDH.

The XRD pattern of CoMgAl-LDH hydrotalcite synthesized with different Co : Mg : Al ratios was shown in Fig. 1, which displayed the typical signals of hydrotalcite materials (JCPDS No. 14-0191). The diffraction peaks at 11.5°, 22.9°, 34.7°, 39.1°, and 46.3° were attributed to the (003), (006), (009), (015), and (018) crystal faces, respectively, indicating a well-formed layered crystal structure with rhombohedral symmetry (3*R*). No diffraction peaks of cobalt ions were observed, indicating that cobalt ions had successfully entered the layered structure of hydrotalcite without forming an independent crystal phase.^22^Fig. 2 showed the XRD pattern of CoMgAl-LDO obtained by calcining hydrotalcite at 400 °C. After calcination, the structure of LDH was destroyed, and characteristic peaks of Al_2_O_3_, Co_3_O_4_, and MgO were detected, forming the highly dispersed metal composite oxide.^23^ Among them, the diffraction peaks at 36.9°, 44.8°, and 65.2° belonged to the (311), (400), and (440) crystal faces of Co_3_O_4_ (JCPDS No. 43-1003), respectively. As shown in Fig. 3, the XRD pattern of Ru/CoMgAl-RLDH exhibited peaks corresponding to the (003), (006), (009), (015), and (018) crystal planes. These peaks were characteristic of hydrotalcite (JCPDS No. 14-0191). The obtained XRD patterns convincingly demonstrated that the mixed metal oxide formed from the thermal treatment of hydrotalcite had reverted to its layered-structured hydrotalcite state after undergoing rehydration. In essence, the XRD results confirmed the hydrotalcite's memory effect. The signal of the Co_3_O_4_ phase was visible, indicating that the reconstruction of the hydrotalcite phase was partial.^24^ In comparison with the XRD patterns of CoMgAl-LDH, those of Ru/CoMgAl-RLDH and CoMgAl-LDO exhibited a rougher appearance. This was likely attributed to the reduction in the crystallinity of hydrotalcite as a consequence of calcination and rehydration processes. The diffraction peaks became less distinct and sharp.^25^

**Fig. 4:**
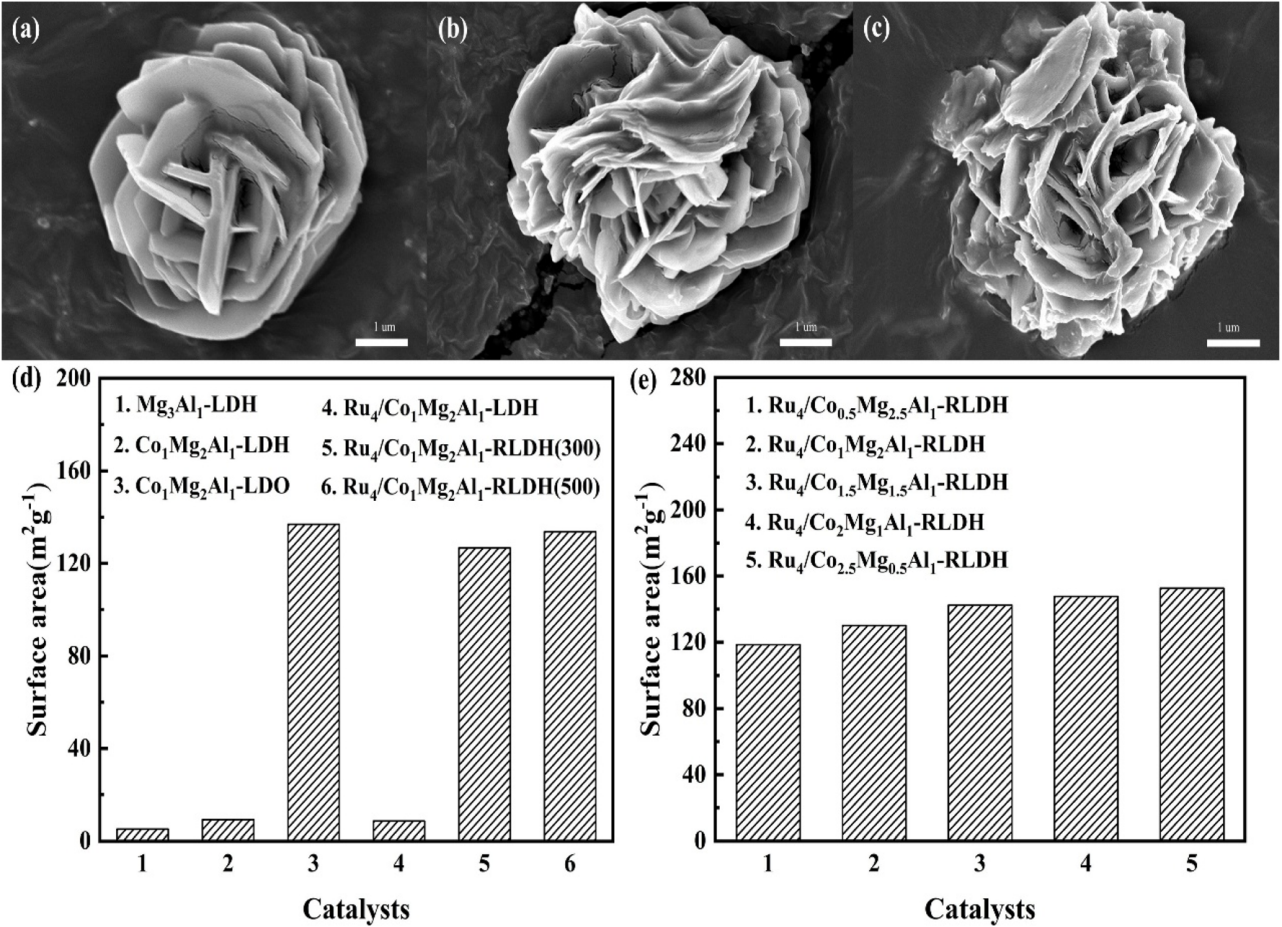
SEM images of (a) Co_1_Mg_2_Al_1_-LDH, (b) Co_1_Mg_2_Al_1_-LDO, (c) Ru_4_/Co_1_Mg_2_Al_1_-RLDH; (d) and (e) Specific surface area of catalysts under different preparation conditions.

The SEM images of Co_1_Mg_2_Al_1_-LDH, Co_1_Mg_2_Al_1_-LDO, and Ru_4_/Co_1_Mg_2_Al_1_-RLDH were presented in Fig. 4. As could be seen from Fig. 4(a), Co_1_Mg_2_Al_1_-LDH exhibited a typical layered structure, with visible stacking arrangements between layers. Fig. 4(b) depicted the SEM image of Co_1_Mg_2_Al_1_-LDO after calcination, revealing a roughened surface due to the disruption of the layered structure and the formation of pores. Fig. 4(c) illustrated the SEM image of Ru_4_/Co_1_Mg_2_Al_1_-RLDH, where the surface remained rough even after calcination and subsequent hydration, likely due to irregular fractures that might have occurred during the rehydration process when the metal oxide was reconstructed into hydrotalcite.

The specific surface area of catalysts prepared under different conditions was tested using N_2_ adsorption–desorption. As shown in Fig. S1,† the N_2_ adsorption–desorption curves of various catalysts conformed to Type IV isotherms, and a typical H3-type hysteresis loop was observed within the relative pressure range of 0.6–1.0(*P*/*P*_0_), which indicated the presence of slit-like pores formed by the aggregation of flaky particles.

As shown in Fig. 4(d), the specific surface area of Mg_3_Al_1_-LDH was measured to be 5.1 m^2^ g^−1^, whereas that of Co_1_Mg_2_Al_1_-LDH reached 9.2 m^2^ g^−1^. This observation suggested that the incorporation of Co into the hydrotalcite structure had the potential to enhance the specific surface area of hydrotalcite. After undergoing the calcination process, the specific surface area of Co_1_Mg_2_Al_1_-LDO experienced an increase, reaching 136.9 m^2^ g^−1^. During the calcination process, the disordered migration of Al significantly enhanced the layered structure, leading to an increase in interlayer spacing, removal of interlayer anions and water molecules, and formation of numerous pores, resulting in the significant increase in specific surface area. The pore volume of the catalyst Co_1_Mg_2_Al_1_-LDH was 0.0313 cm^3^ g^−1^, and its average pore diameter was 13.53 nm. Following calcination, the pore volume of Co_1_Mg_2_Al_1_-LDO became 0.1124 cm^3^ g^−1^, while the average pore diameter was 3.28 nm. As illustrated in Fig. 4(e), when the hydrotalcite underwent rehydration to form Ru_4_/Co_1_Mg_2_Al_1_-RLDH, the specific surface area of the catalyst diminished to 130.0 m^2^ g^−1^. However, the specific surface area of the calcined and rehydrated hydrotalcite was still higher than that of the original hydrotalcite. This was because, although the calcined oxide could recover its original layered structure after rehydration, the crystallinity and orderliness around Al were not fully restored. After rehydration, the pore volume of the catalyst Ru_4_/Co_1_Mg_2_Al_1_-RLDH was 0.1743 cm^3^ g^−1^, and the average pore diameter was 5.36 nm. The specific surface area of Ru_4_/Co_0.5_Mg_2.5_Al_1_-RLDH was 118.5 m^2^ g^−1^. As the Co content in the catalyst increased, the specific surface area gradually increased. When the Co : Mg : Al ratio was 2.5 : 0.5 : 1, the specific surface area of the catalyst Ru_4_/Co_2.5_Mg_0.5_Al_1_-RLDH increased to 152.6 m^2^ g^−1^. This might have been due to the fact that during the preparation of the catalyst, an appropriate amount of cobalt was calcined into oxide without being reconstructed into hydrotalcite, further causing an upward trend in the specific surface area. This corresponded to the appearance of Co_3_O_4_ diffraction peaks in the XRD pattern of the reconstructed hydrotalcite. The specific surface area of Ru_4_/Co_1_Mg_2_Al_1_-RLDH (300) was 126.7 m^2^ g^−1^ at the calcination temperature of 300 °C, 130.0 m^2^ g^−1^ at 400 °C, and 133.7 m^2^ g^−1^ at 500 °C. As the calcination temperature increased, the specific surface area of the catalyst slightly increased, while the average pore diameter gradually decreased. This might have been attributed to the decreasing degree of recovery of the reconstructed hydrotalcite with increasing calcination temperature (Table S1†).

The FTIR spectra exhibited characteristic bands of Co_1_Mg_2_Al_1_-LDH, Co_1_Mg_2_Al_1_-LDO and Ru_4_/Co_1_Mg_2_Al_1_-RLDH (Fig. S2†). The band at 3500 cm^−1^ corresponded to the stretching vibration of OH groups. A shoulder peak observed at 3000 cm^−1^ was due to hydrogen bonding between H_2_O and CO_3_^2−^ in the interlayer. The band at 1650 cm^−1^ arose from the bending vibration (angular deformation) of water molecules. The band at 1500 cm^−1^ was attributed to the vibration caused by the reduced symmetry of interlayer CO_3_^2−^ ions due to the interaction between metal cations and oxygen atoms of carbonate. The band at 1370 cm^−1^ was observed from the typical vibration mode of CO_3_^2−^ ions. All bands below 800 cm^−1^ were ascribed to metal–oxygen interactions. However, due to the overlap of broad absorption bands, it was difficult to assign each band to the corresponding metal–oxygen interaction.^26^ The peak at 3500 cm^−1^ was baseline-corrected and deconvoluted for peak fitting to calculate the peak area. The peak area was recovered to 71.2% of the original value, indicating that the reconstruction degree of hydrotalcite was approximately 71.2%.

### 3.2. Surface chemical evaluation of catalysts

During the CO_2_-TPD measurements, carbonate ions were released from the interlayers of hydrotalcite. Thus, CO_2_-TPD was not suitable for assessing the basic properties of hydrotalcite. Therefore, this study employed Hammett indicator titration for qualitative and quantitative analysis of the fundamental properties of hydrotalcite.^15^ This approach was extensively employed to conduct a macroscopic analysis of the basic properties of catalysts, relying on the color changes that were dependent on the pH value. Bromothymol blue was used to confirm the overall quantity of basic sites. These sites are categorized into strong and weak bases. To analyze medium-strong and strong basic sites, phenolphthalein indicator and golden lotus orange O indicator are employed, respectively. The quantity of weak basic sites was obtained by deducting the number of strong basic sites from the overall count of basic sites.

As shown in Table 1, bromothymol blue, phenolphthalein, and golden lotus orange O were utilized as Hammett indicators for the qualitative analysis of the basic center strength of hydrotalcite. The catalyst was accurately weighed at 25 mg. Subsequently, 1.00 mL of methanol solution with a concentration of 0.1 wt% was introduced. Ultrasonic oscillation was applied, and adsorption equilibrium was maintained for 2 h. The change in hue of the solution was noticed. If the solution exhibited an alkaline color, it indicated that the basicity of the catalyst was stronger than that of the indicator; conversely, if the solution displayed an acidic color, it suggested that the basicity of the catalyst was weaker than that of the indicator. The potency of the basic sites was qualitatively examined by taking note of the color of the solution.

**Table 1:** Hammett indicator method

Indicator	Variation in color		p*K*_BH_^+^
	Acidic color	Alkaline color	
Bromothymol blue	Yellow	Blue	7.3
Phenolphthalein	Colorless	Magenta	9.5
Golden lotus orange O	Yellow	Orange	11.1

To quantitatively analyze the alkali content of the catalyst, this study employed benzoic acid titration to determine the Brønsted alkali content of the catalyst. Initially, 0.1 g of the catalyst was accurately weighed and added to 10.00 mL of methanol. Subsequently, 3 to 4 drops of indicator-methanol solution with a concentration of 0.1 wt% were added and the mixture was thoroughly shaken. The solution was then titrated with benzoic acid–methanol solution whose concentration was 0.01 mol L^−1^, followed by ultrasonic oscillation to facilitate adsorption equilibrium for 2 h. The titration endpoint was reached when the indicator adsorbed on the catalyst surface transitioned from an alkaline to an acidic color. The formula given hereinafter could be utilized to calculate the alkali content of the catalyst.
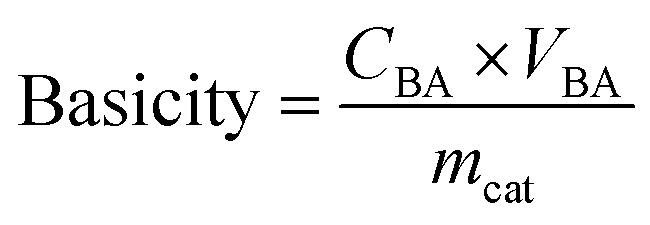
Herein, basicity referred to the alkali content of the catalyst, mmol g^−1^; *C*_BA_ represented the concentration of benzoic acid, mol L^−1^; *V*_BA_ denoted the volume of benzoic acid that was consumed in the course of the titration process, mL; *m*_cat_ denoted the mass of the catalyst, g.

This study employed the Hammett indicator method to measure the basicity of different catalyst surfaces, with the results presented in Table 2. After the incorporation of cobalt to prepare Co_1_Mg_2_Al_1_-LDH, the overall amount of basic active centers on the surface of the catalyst declined. The total basicity of the Co_1_Mg_2_Al_1_-LDH catalyst surface was 0.25 mmol g^−1^, all of which were weak basic sites. The total basicity of the Ru_4_/Co_1_Mg_2_Al_1_-LDH catalyst surface was 0.24 mmol g^−1^, and it also consisting entirely of weak basic sites. After calcination and subsequent rehydration, the total basicity of the Ru_4_/Co_1_Mg_2_Al_1_-RLDH catalyst surface reached 0.37 mmol g^−1^, with 0.21 mmol g^−1^ of weak basic sites and 0.16 mmol g^−1^ of moderate-to-strong basic sites. The data revealed that the original hydrotalcite generally only possessed weak basic sites. However, after calcination and subsequent rehydration, the hydrotalcite exhibited both weak and moderate-to-strong basic sites, providing the richer array of basic sites for reactions.^15^ This was attributed to the calcination and subsequent rehydration, which fully exposed the center of the hydrotalcite structure, revealing moderate-to-strong basic sites. The basic sites of the reconstructed hydrotalcite primarily originated from weak basic sites (OH-functional groups) and moderate-to-strong basic sites (Mg–O pairs).^27^ The original hydrotalcite, after calcination and subsequent rehydration, could obtain the richer array of basic sites. In the reaction, the catalyst Ru_4_/Co_1_Mg_2_Al_1_-RLDH exhibited the higher yield of FDCA compared to Ru_4_/Co_1_Mg_2_Al_1_-LDH, indicating that calcination followed by rehydration enhanced the activity of the catalyst (Table S2†).

**Table 2:** Determination of the alkalinity of catalysts

Catalysts	Base strength	Total (mmol g^−1^)	Strong (mmol g^−1^)	Weak (mmol g^−1^)
Mg_3_Al_1_-LDH	7.3	0.31	—	0.31
Co_1_Mg_2_Al_1_-LDH	7.3	0.25	—	0.25
Ru_4_/Co_1_Mg_2_Al_1_-LDH	7.3	0.24		0.24
Ru_4_/Co_0.5_Mg_2.5_Al_1_-RLDH	9.5	0.41	0.18	0.23
Ru_4_/Co_1_Mg_2_Al_1_-RLDH	9.5	0.37	0.16	0.21
Ru_4_/Co_1.5_Mg_1.5_Al_1_-RLDH	9.5	0.33	0.12	0.21
Ru_4_/Co_2_Mg_1_Al_1_-RLDH	9.5	0.26	0.07	0.19
Ru_4_/Co_2.5_Mg_0.5_Al_1_-RLDH	9.5	0.18	0.05	0.13
Ru_4_/Co_1_Mg_2_Al_1_-RLDH(300)	9.5	0.36	0.13	0.23
Ru_4_/Co_1_Mg_2_Al_1_-RLDH(500)	9.5	0.39	0.22	0.16

The surfaces of catalysts constructed from hydrotalcite with varying molar ratios of cobalt, magnesium, and aluminum exhibited distinct amounts of basicity. Specifically, the total basicity on the surface of the Ru_4_/Co_0.5_Mg_2.5_Al_1_-RLDH catalyst reached 0.41 mmol g^−1^, significantly higher than that of other catalysts. As the cobalt content was further increased, the quantity of basic sites on the catalyst surface decreased accordingly. At the Co : Mg : Al ratio of 2.5 : 0.5 : 1, the total basicity on the surface of the Ru_4_/Co_2.5_Mg_0.5_Al_1_-RLDH catalyst decreased to 0.18 mmol g^−1^. This might have been attributed to the fact that hydrotalcite surfaces constructed with transition metals, exhibited fewer basic sites compared to those constructed with alkaline earth metals.^28^ Among the catalysts with varying molar ratios of cobalt, magnesium, and aluminum, the Ru_4_/Co_1_Mg_2_Al_1_-RLDH catalyst achieved the highest yield in the selective oxidation of HMF to FDCA, indicating that the incorporation of an appropriate amount of cobalt facilitated the selective conversion of HMF to FDCA through oxidation (Table S2†).

When the calcination temperature was 300 °C, the total alkali amount on the surface of the Ru_4_/Co_1_Mg_2_Al_1_-RLDH (300) catalyst was 0.36 mmol g^−1^. At the calcination temperature of 400 °C, the total alkali amount on the surface of the Ru_4_/Co_1_Mg_2_Al_1_-RLDH catalyst was 0.37 mmol g^−1^. When the calcination temperature reached 500 °C, the total alkali amount on the surface of the Ru_4_/Co_1_Mg_2_Al_1_-RLDH (500) catalyst was 0.39 mmol g^−1^. As the calcination temperature increased, the total alkaline sites present on the surface of the catalyst slightly increased, while the moderate-to-strong alkali amount initially rose from 0.13 mmol g^−1^ to 0.16 mmol g^−1^, and then further climbed to 0.22 mmol g^−1^ at the calcination temperature of 500 °C. The alkali strength distribution and alkaline sites of hydrotalcite could be designed by controlling the calcination temperature. When the Ru_4_/Co_1_Mg_2_Al_1_-RLDH(300) catalyst was calcined at 300 °C, the yield of FDCA was 57.6%. When the catalyst was calcined at 400 °C, FDCA was obtained with the yield of 65.1%. However, the yield of FDCA reached its maximum value of 50.9% when the catalyst was calcined at 500 °C, indicating that an appropriate calcination temperature was beneficial to the activity of the catalyst (Table S2†).

The alkaline sites of hydrotalcite could be systematically engineered through calcination and rehydration, and the doping of Co also influenced the alkaline sites on the surface of hydrotalcite. Rational design of the alkaline sites of hydrotalcite was conducive to the transformation of HMF into FDCA through oxidation.

### 3.3. Valence state of catalyst

**Fig. 5:**
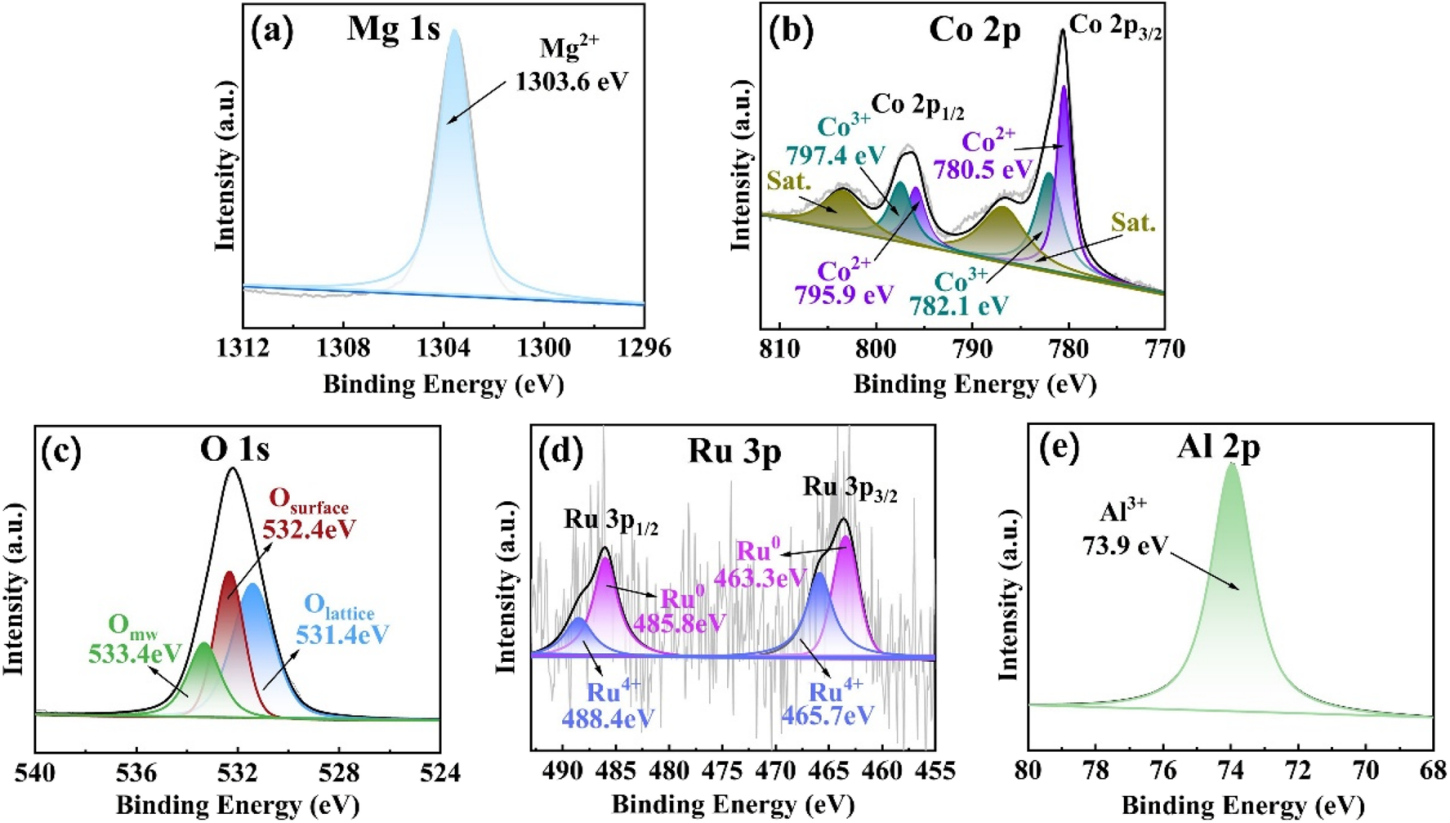
XPS spectra of Ru_4_/Co_1_Mg_2_Al_1_-RLDH, showing (a) Mg 1s, (b) Co 2p, (c) O 1s, (d) Ru 3p and (e) Al 2p.

Fig. 5(a) revealed the Mg 1s peak centered at 1303.6 eV, signifying that Mg existed on the catalyst in the form of Mg^2+^. Fig. 5(b) revealed the XPS spectrum of Co 2p, which had two significant peaks at 780.6 eV and 796.5 eV. These peaks were respectively associated with Co 2p_3/2_ and Co 2p_1/2_. The magnitude of the spin–orbit splitting for the energy levels of Co 2p_3/2_ and Co 2p_1/2_ was 15.9 eV, suggesting the coexistence of Co elements in valence states of Co^2+^ and Co^3+^ in the catalyst. The fitted peaks at energy values of 780.5 eV and 795.9 eV were related to the characteristic peaks associated with Co^2+^ in the Co 2p_3/2_ and Co 2p_1/2_ orbitals respectively. Meanwhile, the fitted peaks at energy values of 782.1 eV and 797.4 eV were linked to the characteristic peaks corresponding to Co^3+^ in the Co 2p_3/2_ and Co 2p_1/2_ orbitals respectively.^29^Fig. 5(c) showed the XPS analysis spectrum of O 1s. The fitted peak at 531.4 eV was designated as O_lattice_, the one at 533.4 eV was named O_mw_, and the peak at 532.4 eV was termed O_surface_. The O_lattice_ peak was attributed to “O^2−^” in lattice oxygen, the O_surface_ peak to oxygen species “O^−^” and “O_2_^2−^” adsorbed on the catalyst surface, and the O_mw_ peak to adsorbed water molecules. Among these three types of oxygen species, O_lattice_, as a nucleophilic reagent, could react with adsorbed hydrogen formed by dehydrogenation of hydroxyl groups in HMF to generate water, facilitating the selective oxidation reaction. O_surface_, with higher mobility, was easily activated and also contributed to enhancing catalytic activity.^30^ The abundance of oxygen species in Ru_4_/Co_1_Mg_2_Al_1_-RLDH was one of the reasons for the catalyst's efficient oxidation of HMF. Fig. 5(d) showed the XPS spectrum of Ru 3p. The peaks with energy values of 463.6 eV and 485.9 eV were assigned to Ru 3p_3/2_ and Ru 3p_1/2_ respectively. The energy difference due to spin–orbit splitting between these two peaks was around 22.3 eV, suggesting the co-existence of Ru^0^ and Ru^4+^ species on the surface of the material. The peaks obtained through fitting, at energy levels of 463.3 eV and 485.8 eV, were respectively related to the Ru^0^ characteristic peaks in the Ru 3p_3/2_ and Ru 3p_1/2_ orbitals. Similarly, the peaks from fitting at 465.7 eV and 488.4 eV were respectively associated with the Ru^4+^ characteristic peaks in the Ru 3p_3/2_ and Ru 3p_1/2_ orbitals.^31^ The existence of Ru^0^ could facilitate the selective oxidation of HMF for the synthesis of FDCA. Moreover, Ru^0^ served as the active center within Ru_4_/Co_1_Mg_2_Al_1_-RLDH.^32^ During the reaction process, Ru^0^ present on the surface of the catalyst was oxidized to Ru^4+^, which might have been a primary cause for the deactivation of supported Ru-based catalysts in catalytic reactions. Fig. 5(e) showed that the central peak of Al 2p was located at 73.9 eV, which indicated the presence of Al in the catalyst in the form of Al^3+^.

As shown in Table S3,† the coexistence of Co^3+^ and Co^2+^ peaks confirmed the formation of Co^2+^/Co^3+^ redox electron pairs on the catalyst surface, which facilitated the storage and release of active oxygen.^33^ The binding energy shifts of Ru^0^ on MgAl-LDH and CoMgAl-LDH indicated the electron synergy between Ru and Co. Under the same reaction conditions, the FDCA yield of the Ru_4_/Co_1_Mg_2_Al_1_-RLDH catalyst was higher than that of Ru_4_/Mg_3_Al_1_-RLDH, demonstrating that the synergy between Ru and Co effectively promoted the catalytic activity (Table S2†).

### 3.4. Evaluation of catalyst activity

To investigate the influence of the Co : Mg : Al ratio on catalytic activity, catalysts with varying molar ratios of Co : Mg : Al were prepared and Ru nanoparticles were loaded onto them for application in the HMF oxidation reaction. As shown in Table S2,† as the Co : Mg : Al ratio was altered from 0.5 : 2.5 : 1 to 1 : 2:1, the yield of FDCA increased from 54.9% to 65.1%. However, as the CoMg ratio was further increased, the yield of FDCA declined to 44.7%. This indicated that doping with an appropriate amount of cobalt promoted the selective oxidation process through which HMF was transformed into FDCA.

To ascertain the optimal calcination temperature for the catalyst, the catalyst was subjected to calcination at 300 °C, 400 °C, and 500 °C for 4 h each. Notably, when the catalyst was calcined at 400 °C, the 100% conversion rate of HMF and the 65.1% yield of FDCA were achieved. At calcination temperatures of 300 °C and 500 °C, the HMF conversion rate remained at 100%, but the yield of FDCA decreased to 57.6% and 50.9%, respectively. In contrast, the FDCA yield of the Ru_4_/Co_1_Mg_2_Al_1_-LDH catalyst, which had not undergone calcination and subsequent rehydration, was only 45.8%. This suggested that the calcination and subsequent rehydration of the hydrotalcite structure at an appropriate temperature might have led to the reconstruction of water-binding sites, potentially resulting in stronger or more abundant alkaline sites.^15^

When the noble metal Ru was not loaded and Co_1_Mg_2_Al_1_-LDH was used as the catalyst, HMF achieved the conversion rate of 40.7%, with the yield of FDCA being 19.2%. Employing Co_1_Mg_2_Al_1_-LDO and Co_1_Mg_2_Al_1_-RLDH as catalysts led to an increase in both the conversion rate of HMF and the yield of FDCA. This indicated that the activity of the catalyst obtained after calcination and subsequent hydration was greater than that of the original hydrotalcite Co_1_Mg_2_Al_1_-LDH and the mixed oxide Co_1_Mg_2_Al_1_-LDO.

Co_1_Mg_2_Al_1_-RLDH was selected as the support to conduct further research on how the loading amount affects catalytic activity. When the loading amount was 1 wt%, HMF achieved the conversion rate of 75.4%, with FDCA yielding 39.7%. When the loading amount increased to 2 wt%, HMF reached the conversion rate of 96.1%, and the yield of FDCA stood at 45.8%. At the loading level of 3 wt%, HMF achieved the complete conversion rate of 100%, while the yield of FDCA stood at 52.3%. This indicated that increasing the Ru loading enhanced the catalyst's activity (Table S4†). Among them, the catalyst Ru_4_/Co_1_Mg_2_Al_1_-RLDH exhibited optimal performance at the Ru loading of 4 wt%, achieving the FDCA yield of 65.1%. As the Ru loading continued to rise further, the FDCA yield decreased, possibly due to the agglomeration of catalyst particles caused by excessive Ru loading.^34^ The catalytic performance of the catalysts significantly improved after Ru loading, which highlighted the importance of Ru in the HMF oxidation reaction and its role as the active center of the catalyst.

**Fig. 6:**
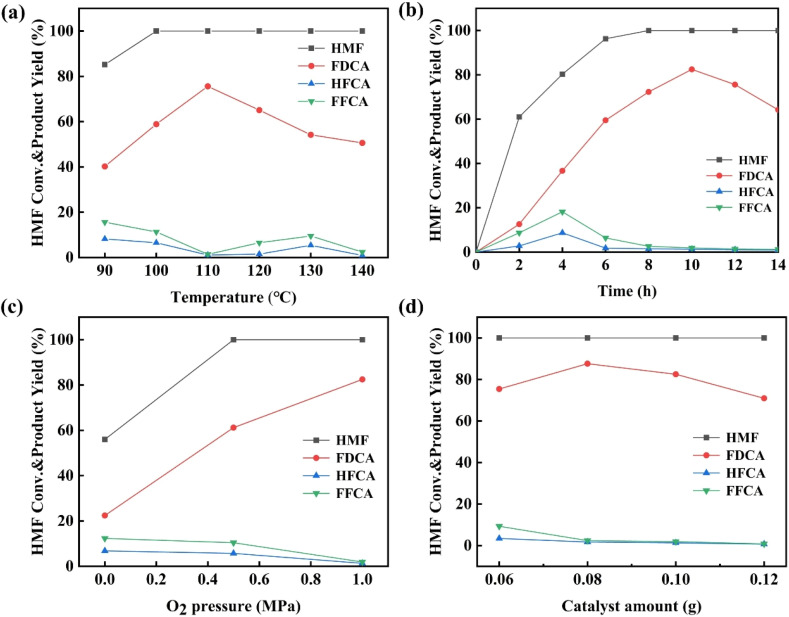
The influence of reaction conditions on the oxidative reaction of HMF. (a) The effect of reaction temperature under the following conditions: 0.10 g Ru_4_/Co_1_Mg_2_Al_1_-RLDH, 1.0 MPa O_2_, and 12 h. (b) The effect of reaction time under the following conditions: 0.10 g Ru_4_/Co_1_Mg_2_Al_1_-RLDH, 1.0 MPa O_2_, and 110 °C. (c) The effect of oxygen pressure under the following conditions: 0.10 g Ru_4_/Co_1_Mg_2_Al_1_-RLDH, 10 h, and 110 °C. (d) The effect of the amount of catalyst under the following conditions: 1.0 MPa O_2_, 10 h, and 110 °C.

Subsequently, the Ru_4_/Co_1_Mg_2_Al_1_-RLDH catalyst was employed to investigate the optimal reaction conditions for the oxidative process that synthesizes FDCA using 0.2 mmol of HMF in 5 mL of aqueous solution. Fig. 6(a) depicts the influence of different temperatures on the selective oxidation involving HMF. When the reaction temperature was 90 °C, HMF conversion stood at 85.2%, with FDCA yield at 40.2%. As the reaction temperature reached 100 °C, HMF achieved complete conversion, and FDCA yield reached 58.9%. At the reaction temperature of 110 °C, the maximum yield of FDCA was 75.6%, and the yields of by-products FFCA and HFCA reached their lowest levels. As the temperature continued to rise, the yield of FDCA gradually decreased, while the yields of by-products FFCA and HFCA first increased and then decreased. Moderate increases in reaction temperature are beneficial for the progress of the reaction, but further increases in temperature led to a decrease in the yield of FDCA which was the targeted product. Continuing to increase the temperature might result in the degradation of FDCA into humic substances.^35^ Therefore, the optimal reaction temperature was 110 °C. The Turnover Number (TON) and Turnover Frequency (TOF) were important criteria for evaluating the performance of catalysts. Based on how the Ru_4_/Co_1_Mg_2_Al_1_-RLDH catalyst performed catalytically at different reaction temperatures, the TON and TOF values were calculated. As can be seen from Table S5,† with increasing reaction temperature, the TON and TOF values also continuously increased. At the reaction temperature of 110 °C, the TON and TOF values reached their maximum, which might have been attributed to the continued reaction of FDCA to generate humic substances or other oxidation products through ring-opening reactions as the temperature increased.^36,37^

Fig. 6(b) illustrated the conversion rate of HMF and the yield of various products as the function of the reaction time. At the reaction time of 2 h, HMF achieved the conversion rate of 61%, while FDCA attained the yield of 12.6%, and the yields of by-products HFCA and FFCA were 2.8% and 8.7%, respectively. At the reaction time of 4 h, HMF conversion rate reached 80.3%, the FDCA yield increased to 36.7%, and the yields of HFCA and FFCA rose to 8.7% and 18.2%, respectively. With the continuous increase of the reaction time up to 10 h, HMF was completely converted, the yield of FDCA reached the maximum of 82.5%, and the yields of by-products HFCA and FFCA decreased to 1.3% and 1.9%, respectively. When the reaction time was further extended to 14 h, the yield of FDCA continued to decline to 64.2%. Prolonged reaction durations beyond an optimal threshold could potentially induce side reactions, including the ring-opening and polymerization of FDCA.^38^ Therefore, the optimal reaction time was 10 h. During the reaction process, DFF was not detected. Thus, it was speculated that the pathway for the oxidation of HMF to synthesize FDCA was HMF → HFCA → FFCA → FDCA.

Oxygen (O_2_) was environmentally friendly and characterized by mild reaction conditions, thus it was selected as the oxidant for the oxidation reaction. Fig. 6(c) illustrated the impact of varying O_2_ pressure conditions on the oxidation of HMF. As the reaction pressure increased, the yield of FDCA also rose. At normal oxygen pressure, the HMF conversion rate was 56%, the FDCA yield was only 22.4%, and the yields of by-products HFCA and FFCA were 6.8% and 12.3%, respectively. As the reaction pressure increased to 0.5 MPa, complete conversion of HMF was achieved, the FDCA yield increased to 61.2%, and the yields of by-products HFCA and FFCA decreased to 5.7% and 10.4%, respectively. Once the oxygen pressure reached 1.0 MPa, the complete conversion of HMF was realized and the yield of FDCA hit 82.5%. At the same time, the yields of by-products HFCA and FFCA fell to 1.3% and 1.9% respectively. The oxygen concentration was closely related to the oxygen partial pressure. In this reaction system, the higher oxygen concentration led to more oxygen present at the active sites, thereby enhanced the FDCA yield.^39^ Therefore, an oxygen pressure of 1.0 MPa was the optimal reaction gas atmosphere, and this reaction pressure would be used for subsequent studies.

Fig. 6(d) illustrated the impact of varying catalyst dosages on the process of selectively oxidizing HMF into FDCA. An increase or decrease in catalyst dosage directly modified the quantity of catalytically active sites, thus having an impact on the reactivity of the reaction. When 0.06 g of Ru_4_/Co_1_Mg_2_Al_1_-RLDH was introduced into the reaction system, HMF was completely converted, achieving the FDCA yield of 75.4%, with by-product yields of HFCA and FFCA being 3.4% and 9.3%, respectively. This demonstrated the excellent catalytic activity of Ru_4_/Co_1_Mg_2_Al_1_-RLDH in the oxidation of HMF. With the further increase in the catalyst dosage to 0.08 g, the FDCA yield rose to 87.6%, while the yields of by-products HFCA and FFCA decreased to 1.7% and 2.4%, respectively. However, as the catalyst dosage continued to increase, the FDCA yield began to decline. When the catalyst dosage reached 0.12 g, the FDCA yield dropped to 70.9%, with by-product yields of HFCA and FFCA merely 0.7% and 0.8%, respectively. This could be due to the over-oxidation of HMF into other by-products like levulinic acid, formic acid, humic acid, and glycolic acid.^35^ In summary, 0.08 g of Ru_4_/Co_1_Mg_2_Al_1_-RLDH provided suitable catalytic sites for the reaction, thus indicated that the optimal catalyst dosage was 0.08 g.

### 3.5. The cyclic stability of the catalyst

**Fig. 7:**
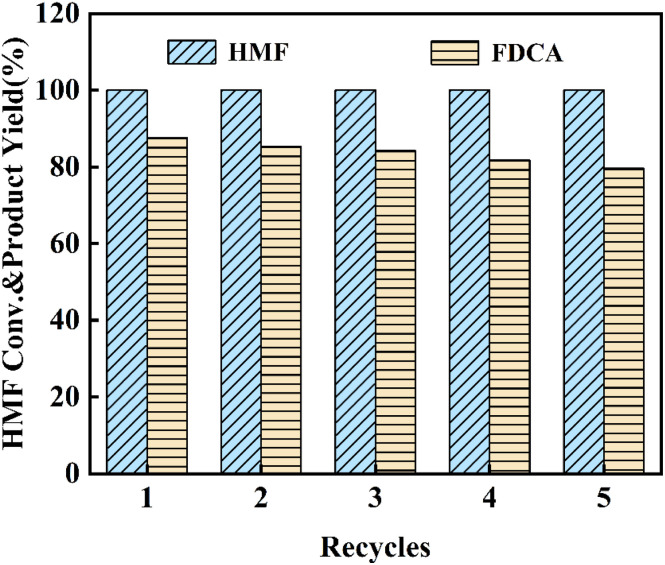
Cyclic stability of Ru_4_/Co_1_Mg_2_Al_1_-RLDH under optimal conditions.

For the industrial utilization of catalysts, their stability and recyclability represent vital evaluation criteria. Under optimal reaction conditions, a cyclic experiment was conducted on the Ru_4_/Co_1_Mg_2_Al_1_-RLDH catalyst for the oxidation. Following the reaction, distilled water and ethanol were used to wash the catalyst. Subsequently, it was dried at 80 °C for 12 h under vacuum conditions. As depicted in Fig. 7, after five repetitions, HMF conversion rate held steady at 100%, and the yield of FDCA declined to 79.4%, indicating good activity retention.

**Fig. 8:**
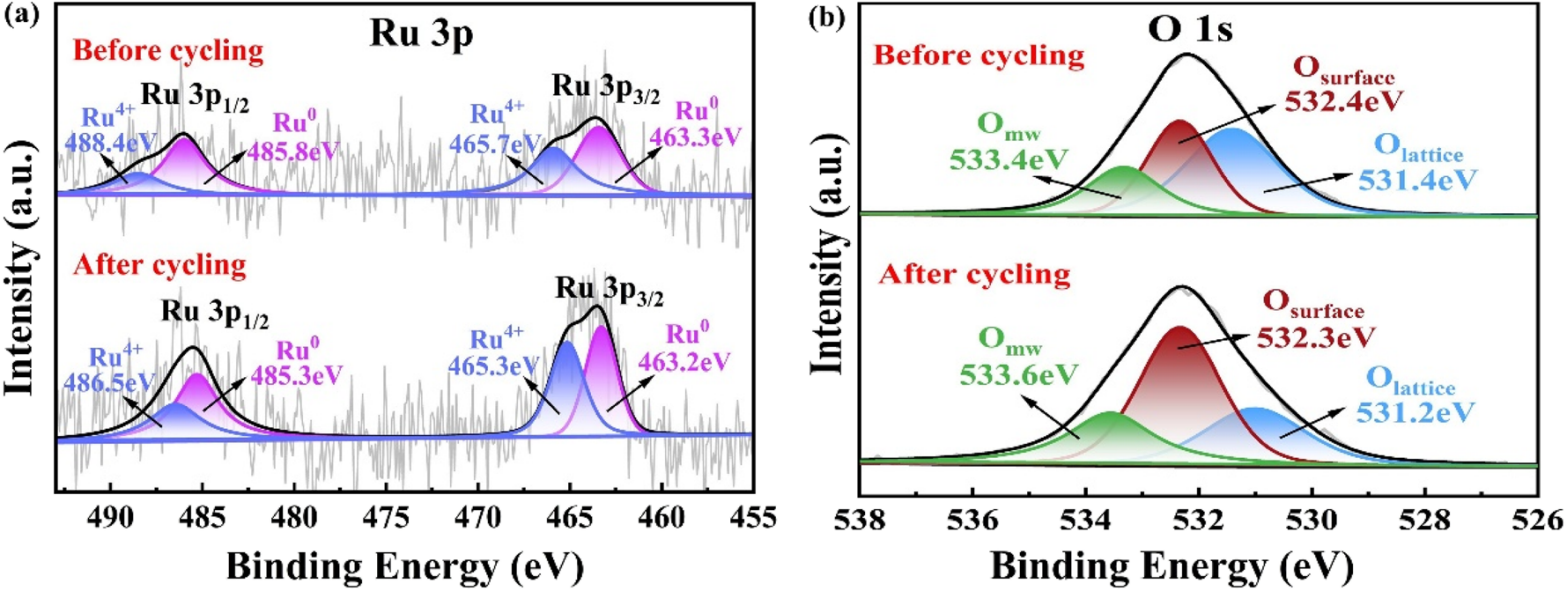
XPS spectra of Ru_4_/Co_1_Mg_2_Al_1_-RLDH before and after cyclic experiments: (a) Ru 3p and (b) O 1s.

Fig. 8 presented the XPS spectra of Ru 3p and O 1s for Ru_4_/Co_1_Mg_2_Al_1_-RLDH both before use and after five cycles of repeated use. Following five cycles of repeated use, the proportion of Ru^0^ in the catalyst Ru_4_/Co_1_Mg_2_Al_1_-RLDH decreased from 58.5% to 55.3%, indicating that Ru^0^ was partially oxidized to Ru^4+^ during the cycling process. The oxidation of Ru^0^ on the catalyst surface was commonly attributed to the reduced activity of the Ru species supported on the catalyst in the oxidation reactions of HMF. After five cycles of repeated use, the O_lattice_ species on the surface of the catalyst Ru_4_/Co_1_Mg_2_Al_1_-RLDH were consumed during the HMF oxidation reaction, with O_lattice_ decreasing from 45.4% to 26.5%. During the reaction process, O_2_ molecules dissociated into surface adsorbed oxygen species on the catalyst surface, resulting in an increase in O_surface_ from 32.3% to 47.8%. This showed that O_lattice_ was essential in the course of HMF oxidation.^40^

**Fig. 9:**
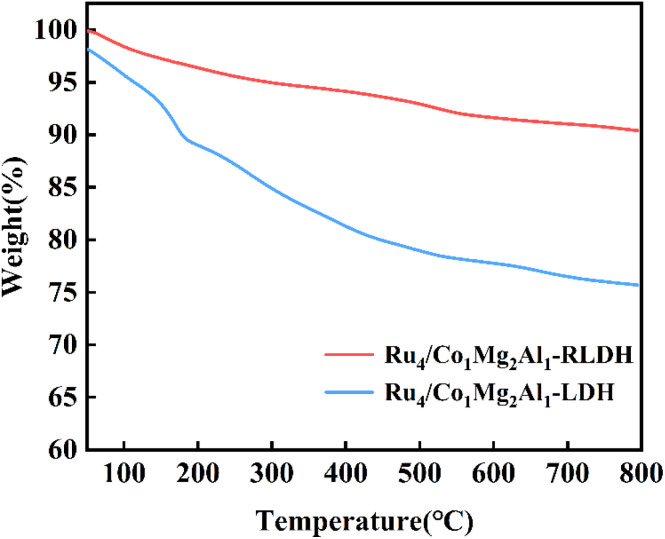
Thermogravimetric curve of Ru_4_/Co_1_Mg_2_Al_1_-RLDH and Ru_4_/Co_1_Mg_2_Al_1_-LDH.

Fig. 9 illustrated the thermogravimetric analysis (TGA) of Ru_4_/Co_1_Mg_2_Al_1_-RLDH and Ru_4_/Co_1_Mg_2_Al_1_-LDH, which examined the weight loss of these catalysts as the temperature increased. During the heating process, the reduction in the catalysts' mass below 250 °C was primarily due to the evaporation of interlayer water and physically adsorbed water in hydrotalcite, a process that did not affect the layered hydroxide structure. The weight loss of the catalysts between 250 °C and 450 °C mainly corresponded to the dehydroxylation of the layered plates and the decomposition of some interlayer carbonate ions. The weight loss of the catalysts between 450 °C and 800 °C primarily corresponded to the decomposition of interlayer anions.^41^ During the reconstruction process, although some water molecules and anions could re-enter the interlayer, their content was lower than that in the original hydrotalcite, resulting in around 10% of the weight being lost for the calcined hydrotalcite Ru_4_/Co_1_Mg_2_Al_1_-RLDH at 800 °C, compared to around 25% of the weight being lost for the original hydrotalcite Ru_4_/Co_1_Mg_2_Al_1_-LDH. This indicated that Ru_4_/Co_1_Mg_2_Al_1_-RLDH exhibited better stability than the original hydrotalcite.

## 4. Reaction mechanism

**Fig. 10:**
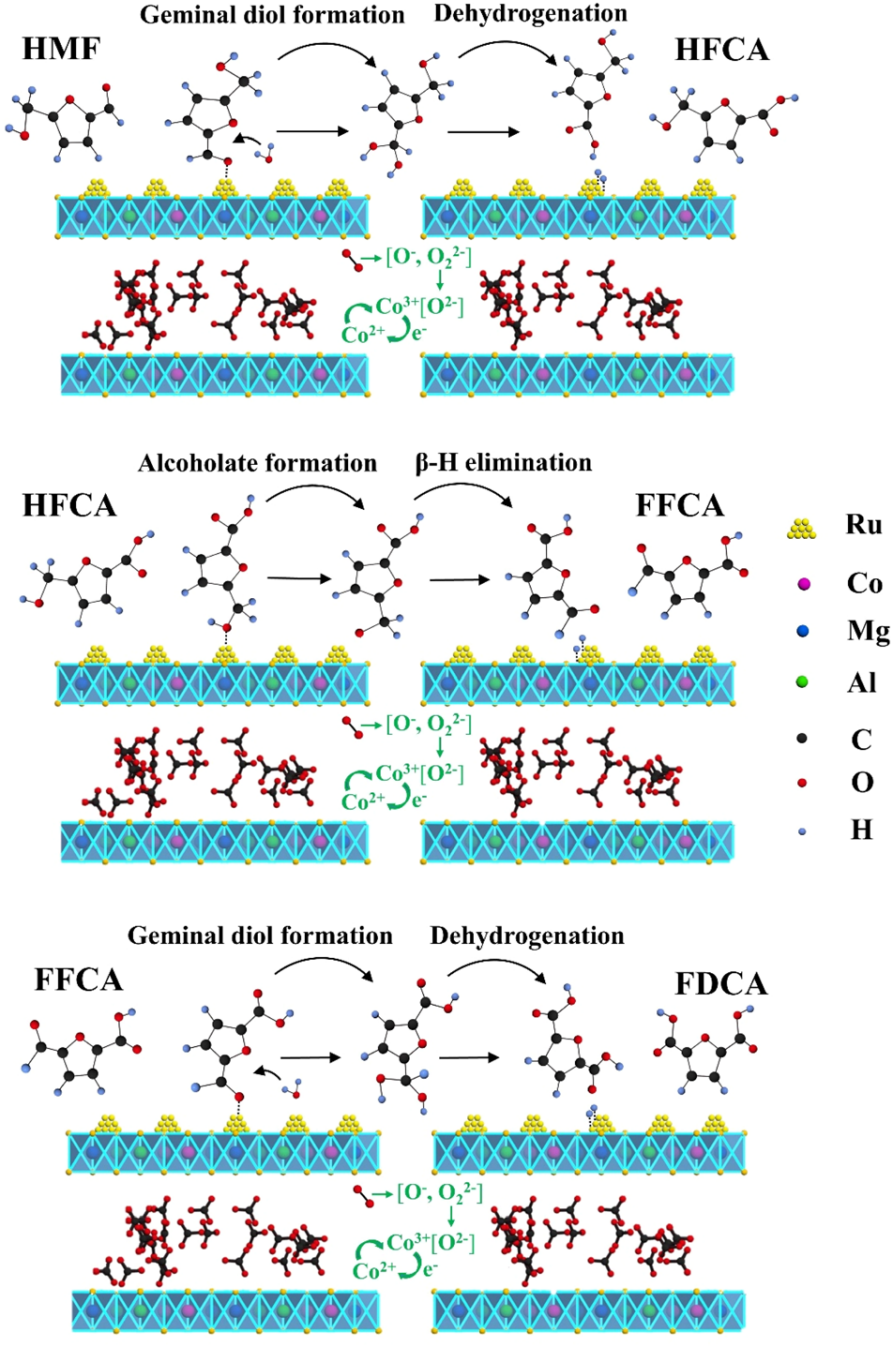
Possible reaction mechanism for the catalytic oxidation of HMF to FDCA facilitated by Ru_4_/Co_1_Mg_2_Al_1_-RLDH.

In light of the experimental findings described previously, a potential reaction mechanism for the Ru_4_/Co_1_Mg_2_Al_1_-RLDH catalyst was proposed, as illustrated in Fig. 10. Along this reaction course, the aldehyde group of HMF initially attached to the Ru^0^ active site to undergo hydrolysis, forming an intermediate geminal diol.^42^ The oxygen adsorbed on the catalyst surface was activated and was converted into reactive lattice oxygen at the catalyst support's oxygen vacancies. On Ru^0^, the O–H bond of the intermediate geminal diol broke, generating two adsorbed hydrogen atoms. These hydrogen atoms then reacted with the catalyst surface's active lattice oxygen to form water, thereby finishing the intermediate's dehydrogenation and yielding HFCA.^43^ Subsequently, the hydroxyl group in HFCA was adsorbed onto the catalyst surface, removing one hydrogen atom through the breaking of the O–H bond, forming an intermediate of metal-alkoxide. Then, through β-H elimination, the hydrogen atom attached to the carbon atom was removed, converting the alcohol functionality into an aldehyde-type structure to generate another intermediate product, FFCA.^33^ The aldehyde-containing side chain in FFCA underwent hydrolysis to generate a second intermediate geminal diol. This diol then released two hydrogen atoms that remained on the catalyst surface and reacted with the catalyst-surface active lattice oxygen to produce water, ultimately leading to the formation of the target product, FDCA. Considering the generated intermediate products, the reaction route of this catalyst was surmised to be HMF → HFCA → FFCA → FDCA. During this reaction process, the reduction of Co^3+^ to Co^2+^ consumed surplus electrons present on the catalyst support's surface (Co^3+^ + e^−^ → Co^2+^). O_2_ that was adsorbed onto the catalyst surface dissociated into reactive oxygen species O_2_^2−^ and O^−^, which were then further converted to lattice oxygen O^2−^. Accompanied by the formation of lattice oxygen, Co^2+^ was oxidized to Co^3+^.

The catalytic aerobic oxidation of HMF to FDCA is a complex oxidation process that can generate several intermediates, including HFCA, DFF, and FFCA, depending on factors such as the type of active metal, support material, and preparation method. The aerobic oxidation pathway of HMF on hydrotalcite-supported noble metal catalysts primarily involves the oxidation of the aldehyde group in HMF to a carboxyl group, forming HFCA. Subsequently, the hydroxyl group in HFCA is oxidized to an aldehyde group, generating FFCA, and finally, the aldehyde group in FFCA is oxidized to a carboxyl group to produce FDCA. In contrast, the mechanism facilitated by homogeneous alkali catalysts involves the initial oxidation of the hydroxyl group in HMF to an aldehyde group, forming DFF. One of the aldehyde groups in DFF is then oxidized to a carboxyl group, generating FFCA, which is further oxidized at the remaining aldehyde group to yield FDCA.^44^ This mechanistic difference highlights the significant influence of catalyst types on the reaction pathways.

## 5. Conclusion

Cobalt–magnesium–aluminum hydrotalcite was produced by means of the hydrothermal method, and the “memory effect” of hydrotalcite was utilized to rehydrate the calcined hydrotalcite. The catalyst Ru/CoMgAl-RLDH was prepared by the impregnation-reduction method to load ruthenium nanoparticles. The specific surface area of the original hydrotalcite Co_1_Mg_2_Al_1_-LDH was 9.2 m^2^ g^−1^, while the specific surface area of the reconstructed Ru_4_/Co_1_Mg_2_Al_1_-RLDH increased to 130.0 m^2^ g^−1^. The total alkali amount on the surface of the original hydrotalcite Co_1_Mg_2_Al_1_-LDH catalyst was 0.25 mmol g^−1^, all of which were weak basic sites. The total alkali amount on the surface of the reconstructed hydrotalcite Ru_4_/Co_1_Mg_2_Al_1_-RLDH catalyst was 0.37 mmol g^−1^, with a richer set of basic sites, including weak and moderate basic sites. The specific surface area of the reconstructed hydrotalcite was notably greater compared to the original hydrotalcite, and it exhibited higher thermal stability than the original hydrotalcite. The basic sites of the reconstructed hydrotalcite could be systematically adjusted by varying the mole ratio of elements and calcination temperature. In the case where the mole ratio of Co : Mg : Al was 1 : 2 : 1, the calcination temperature was 400 °C, and the Ru loading was 4 wt%, the prepared Ru_4_/Co_1_Mg_2_Al_1_-RLDH catalyst exhibited the highest activity. Under the optimal reaction conditions (110 °C, 10 h, oxygen pressure 1 MPa, 0.08 g catalyst dosage), the conversion rate of HMF was 100%, and the yield of FDCA was 87.6%. After five cycles of experiments, the yield of FDCA slightly decreased to 79.4%. Doping a suitable amount of cobalt into magnesium–aluminum hydrotalcite, calcining and rehydrating the hydrotalcite, and reasonably designing the Ru-loaded reconstructed hydrotalcite were beneficial for the selective oxidation of HMF to FDCA.

## Author contributions

Shuang Zhang: writing–original draft, investigation, conceptualization. Sai Wang: writing–original draft, investigation, conceptualization. Ji Ma: formal analysis, conceptualization. Suzhen Cao: conceptualization.

## Conflicts of interest

There are no conflicts to declare.

## Supplementary Material

RA-015-D5RA02352A-s001

## Data Availability

All the data are provided in the manuscript and ESI† files.
