# Supplementary material for: Reconstruction of cobalt magnesium aluminum hydrotalcite loaded ruthenium using the memory effect for selective oxidation of 5-hydroxymethylfurfural under alkali-free conditions
Source: RSC Adv. 2025 Jul 18;15(31):25413–24. doi: 10.1039/d5ra02352a (PMC12272569; doi:10.1039/d5ra02352a)
Supplement: RA-015-D5RA02352A-s001 [file RA-015-D5RA02352A-s001.pdf]

Supplementary Information (SI) for RSC Advances.
This journal is © The Royal Society of Chemistry 2025

Supporting Information

For

Reconstruction of cobalt magnesium aluminum hydrotalcite loaded ruthenium using memory effect for selective oxidation of 5-hydroxymethylfurfural under alkali free conditions

Shuang Zhang*, Sai Wang, Ji Ma, Suzhen Cao

Institute of Petrochemical Technology, Jilin Institute of Chemical Technology, 45 Chengde Street, Jilin 132022, PR China

*Corresponding author

Corresponding Email: zs3062332@126.com (S.Z.)

Experimental section

Chemicals

5-Hydroxymethylfurfural (HMF, 97%), 2,5-furandicarboxylic acid (FDCA, 98%), and 5-hydroxymethyl-2-furoic acid (HFCA, 97%) were purchased from Shanghai Debo Biochemical Technology Co., Ltd. Sodium borohydride ($NaBH_4$, 97%), cobalt(II) nitrate hexahydrate ($Co(NO_3)_2\cdot 6H_2O$, 99%), magnesium nitrate hexahydrate ($Mg(NO_3)_2\cdot$6H2O, 99%), and aluminum nitrate nonahydrate ($Al(NO_3)_3\cdot 6H_2O$, 99%) were obtained from Sinopharm Chemical Reagent Co., Ltd. Sodium hydroxide (NaOH, 96%) was purchased from Tianjin Damao Chemical Reagent Factory. Ruthenium trichloride trihydrate ($RuCl_3\cdot 3H_2O$, 99%) was bought from Tianjin Xilema Biotechnology Co., Ltd. 5-Formyl-2-furoic acid (FFCA, 98%), methanol (CH_4O, chromatographic grade), formic acid (CH_2O_2, chromatographic grade), and acetonitrile (C_2H_3N, chromatographic grade) were purchased from Shanghai Aladdin Biochemical Technology Co., Ltd. Urea ($CO(NH_2)_2$, 99%), benzoic acid ($C_7H_6O_2$, 99%), bromothymol blue ($C_{27}H_{28}O_5SBr_2$, 95%), phenolphthalein ($C_{20}H_{14}O_4$, 98%), and tropaeolin O ($C_{12}H_9N_2NaO_5S$, 98%) were obtained from Shanghai Macklin Biochemical Technology Co., Ltd.

Catalyst characterizations

Powder X-ray diffraction (XRD) analysis was carried out using a Bruker D8 Advance instrument, with Cu Kα radiation as the radiation source. The operating voltage of this equipment was 40 kV, the scanning range was from 10° to 80°, and the scanning rate was 4°/min.

The N_2 adsorption-desorption analysis was conducted using a Micro for TriStar II Plus 2.02 device. Before the experiment, the sample needed to be degassed in a vacuum environment at 120 °C for 12 h. Subsequently, the adsorption-desorption experiment was carried out at the liquid nitrogen temperature of -196 °C. The specific surface area of the sample was estimated by the Brunauer-Emmett-Teller (BET) formula. Meanwhile, the Barrett-Joyner-Halenda (BJH) technique was employed to calculate its average pore volume and pore size.

The scanning electron microscopy (SEM) was performed using a JEOL-JSM-7610F Plus scanning electron microscope to observe the microscopic morphology of the samples.

The catalyst was analyzed using a TGA/DSC 3+ simultaneous thermogravimetric analyzer. The test was carried out under a nitrogen (N_2) atmosphere. The test temperature ranged from 30 °C to 800 °C, and the heating rate was 2 °C per minute. Each time, the catalyst sample was placed in a thermogravimetric crucible, with a mass of approximately 6 to 8 milligrams.

During the analysis of X-ray photoelectron spectroscopy (XPS), an Escalab 250Xi instrument was employed. This analytical technique utilized Al Kα radiation as the X-ray source to obtain the electronic structure information of the elements on the sample surface. To ensure the accuracy of the analysis results, the binding energy values of all elements were calibrated to 284.8 eV with reference to the peak of carbon (C 1s).

FTIR spectra were obtained using a Thermo Fisher spectrometer in the range of 400-4000 cm^{-1} with a resolution of 2-4 cm^{-1}. Samples were first diluted with KBr powder and then pelletized before introduction to the apparatus.

Preparation of the catalyst $Ru_4/Co_1Mg_2Al_1$-LDH

A total of 12 mmol of $Mg(NO_3)_2·6H_2O$, $Co(NO_3)_2·6H_2O$, and $Al(NO_3)_3·9H_2O$ were dissolved in 30 mL of deionized water at a molar ratio of 2:1:1 to obtain a mixed salt solution. Meanwhile, 24 mmol of urea was dissolved in 30 mL of deionized water. Under stirring conditions, the urea solution was slowly added drop-by-drop to the mixed salt solution. The reaction kettle containing the mixture was subjected to a hydrothermal treatment at 120 °C for 24 h. After the hydrothermal treatment, the mixture was allowed to cool naturally to room temperature. Then it was centrifuged and filtered, and the resulting solid was washed repeatedly with water. The obtained precipitate was dried at 80 °C for 12 h to obtain $Co_1Mg_2Al_1$-LDH.

The Ru active component was loaded by the impregnation-reduction method. A certain amount of $RuCl_3·3H_2O$ (according to a Ru mass fraction of 4 wt% based on the mass of the support) was weighed and dissolved in 12.5 mL of deionized water. Then, 0.5 g of the support CoMgAl-LDH was added to it. Under an ice-water bath and with stirring, the impregnation and dispersion were carried out for 12 h. Sodium borohydride ($NaBH_4$) was weighed according to a molar ratio of BH_4^- in $NaBH_4$ to Ru^{3+} in $RuCl_3·3H_2O$ of 20:1 and dissolved in a 0.5 wt% aqueous NaOH solution to make the concentration of $NaBH_4$ reach 1.5 mol/L. Then, it was dropped into the dispersion of ruthenium trichloride and the support, and the reduction reaction was carried out with stirring for 12 h. The mixed solution was filtered under suction and washed until it was neutral, and then dried in a vacuum for 12 h to obtain the Ru-loaded catalyst, denoted as $Ru_4/Co_1Mg_2Al_1$-LDH.

Product and reaction analysis

The detection wavelengths for HMF, HFCA, FFCA, DFF and FDCA were 284, 250, 287, 288, and 264 nm, respectively.

The conversion ratio of HMF (mol%) can be calculated using the formula (1).

$$\text{The conversion of HMF (mol\%)} = \frac{\text{Moles (HMF converted)}}{\text{Moles (initial HMF)}} \times 100\% \qquad (1)$$

The yield of FDCA (mol%) can be calculated using the formula (2).

$$\text{The yield of FDCA (mol\%)} = \frac{\text{Moles (FDCA produced)}}{\text{Moles (initial HMF)}} \times 100\% \qquad (2)$$

The selectivity of FDCA (mol%) can be calculated using the formula (3).

$$\text{The } selectivity \text{ of FDCA (mol\%)} = \frac{\text{Moles (FDCA produced)}}{\text{Moles (HMF } converted\text{)}} \times 100\% \qquad (3)$$

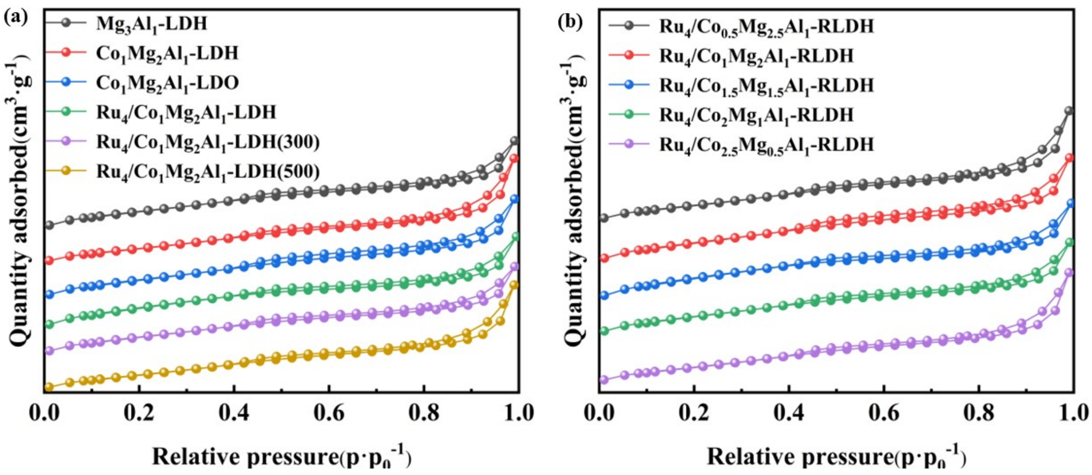

Figure S1. N_2 adsorption-desorption curves of the catalyst

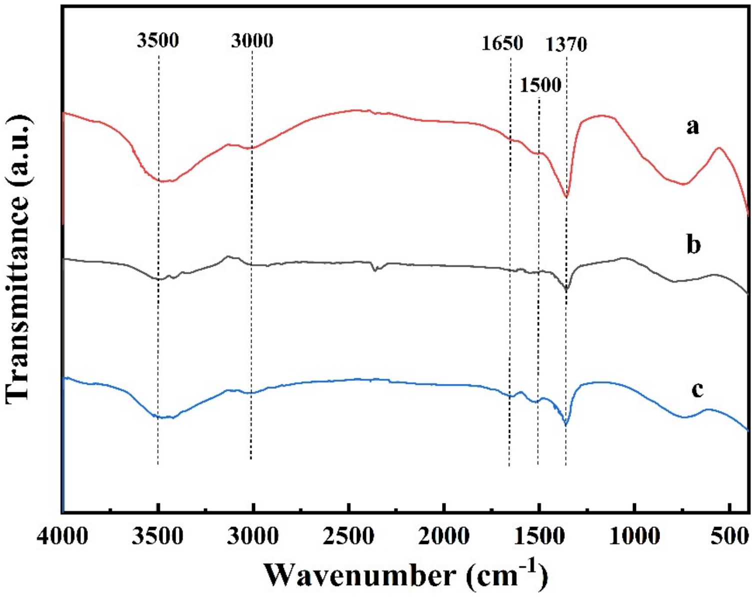

Figure S2. FTIR spectra of (a) $Co_1Mg_2Al_1$-LDH, (b) $Co_1Mg_2Al_1$-LDO and $Ru_4/Co_1Mg_2Al_1$-RLDH.

Table S1. Specific surface area, pore volume and average pore size of the catalyst

Catalyst	Specific surface area (m^2/g)	Pore volume (cm^3/g)	Average pore size (nm)
Mg_3Al_1-LDH	5.1	0.0141	11.19
$Co_1Mg_2Al_1$-LDH	9.2	0.0313	13.53
$Co_1Mg_2Al_1$-LDO	136.9	0.1124	3.28
$Ru_4/Co_1Mg_2Al_1$-LDH	8.6	0.0279	11.97
$Ru_4/Co_{0.5}Mg_{2.5}Al_1$-RLDH	118.5	0.2026	6.84
$Ru_4/Co_1Mg_2Al_1$-RLDH	130.0	0.1743	5.36
$Ru_4/Co_{1.5}Mg_{1.5}Al_1$-RLDH	142.4	0.1974	5.54
$Ru_4/Co_2Mg_1Al_1$-RLDH	147.6	0.1835	5.22
$Ru_4/Co_{2.5}Mg_{0.5}Al_1$-RLDH	152.6	0.1844	4.83
$Ru_4/Co_1Mg_2Al_1$-RLDH(300)	126.7	0.2585	8.47
$Ru_4/Co_1Mg_2Al_1$-RLDH(500)	133.7	0.0979	2.93

Table S2. Screening of catalyst supports for HMF oxidation reaction

Entry	Sample	Conv.(%)	Y_{FDCA}(%)	Y_{FFCA}(%)	Y_{HFCA}(%)
1	Ru_4/Mg_3Al_1-RLDH	100	53.7	1.1	0.9
2	$Ru_4/Co_{0.5}Mg_{2.5}Al_1$-RLDH	100	54.9	2.6	0.9
3	$Ru_4/Co_1Mg_2Al_1$-RLDH	100	65.1	6.5	1.5
4	$Ru_4/Co_{1.5}Mg_{1.5}Al_1$-RLDH	100	52.6	4.5	1.2
5	$Ru_4/Co_2Mg_1Al_1$-RLDH	100	43.6	3.7	1.3
6	$Ru_4/Co_{2.5}Mg_{0.5}Al_1$-RLDH	100	44.7	3.9	1.6
7	$Ru_4/Co_1Mg_2Al_1$-RLDH(300)	100	57.6	2.8	1.2
8	$Ru_4/Co_1Mg_2Al_1$-RLDH(500)	100	50.9	1.7	1.3
9	$Ru_4/Co_1Mg_2Al_1$-LDH	100	45.8	2.3	1.4
10	Mg_3Al_1-LDH	45.1	8.9	1.5	1.9
11	$Co_1Mg_2Al_1$-LDH	40.7	19.2	0.9	1.8
12	$Co_1Mg_2Al_1$-LDO	46.8	25.4	1.1	2.1
13	$Co_1Mg_2Al_1$-RLDH	50.2	28.4	1.5	2.3
14	$Ru_1/Co_1Mg_2Al_1$-RLDH	75.4	39.7	2.8	1.2
15	$Ru_2/Co_1Mg_2Al_1$-RLDH	96.1	45.8	3.3	2.5
16	$Ru_3/Co_1Mg_2Al_1$-RLDH	100	52.3	4.6	2.1
17	$Ru_5/Co_1Mg_2Al_1$-RLDH	100	62.9	1.7	1.3

Reaction conditions: reaction temperature 120 °C, reaction time 12 h, oxygen pressure 1 MPa, catalyst dosage 0.1 g.

Table S3 XPS parameters of the Ru_4/Mg_3Al_1-RLDH and $Ru_4/Co_1Mg_2Al_1$-RLDH catalysts

Catalyst	Binding energy (eV)					
	Ru^0	Co^{2+}	Co^{3+}	Mg^{2+}	Al^{3+}	$O_{lattice}$
Ru_4/Mg_3Al_1-RLDH	462.9	-	-	1303.7	73.7	532.6
$Ru_4/Co_1Mg_2Al_1$-RLDH	463.3	780.5	782.1	1303.6	73.9	531.4

Table S4 Performance comparison on the catalysts for oxidation of HMF to FDCA

Catalyst	Centers	HMF	T/°C	Time/h	Oxidant	Conv./%	Y_{FDCA}/%	Ref.
$Ru/Co_1Mg_2Al_1$-RLDH	4 wt%	0.2 mmol	110	10	1 MPa O_2	100	87.6	this work
PtAu/HT	3 wt%	0.2 mmol	60	12	60 mL/min	100	90	[1]
Au/HT	10 wt%	0.2 mmol	90	7	10 mL/min	100	78	[2]
Au/HT	2 wt%	0.2 mmol	90	7	10 mL/min	100	86	[2]
AuPd/LaCaMgAl-LDH	3 wt%	0.5 mmol	120	6	0.5 MPa	100	99	[3]

Table S5. Performance tests of the catalyst for the oxidation of HMF at different temperatures

Temperature(°C)	Conv.(%)	FDCA Yield(%)	TON[1]	TOF[2](h^{-1})
90	85.2	40.2	2.03	0.17
100	100	58.9	2.97	0.25
110	100	75.6	3.82	0.32
120	100	65.1	3.29	0.27
130	100	54.2	2.74	0.23
140	100	50.6	2.56	0.21

1:Conversion number (Moles of FDCA obtained/Number of active centers),

2:Turnover frequency (TON/Reaction time).

1. H. A. Xia, J. H. An, M. Hong, S. Q. Xu, L. Zhang and S. L. Zuo, Catal. Today, 2019, 319, 113-120.
2. L. Ardemani, G. Cibin, A. J. Dent, M. A. Isaacs, G. Kyriakou, A. F. Lee, C. M. A. Parlett, S. A. Parry and K. Wilson, Chem. Sci., 2015, 6, 4940-4945.
3. Z. Gao, R. F. Xie, G. L. Fan, L. Yang and F. Li, *ACS Sustain. Chem. Eng.*, 2017, **5**, 5852-5861.
